# Evaluation of Cholinesterase Inhibitory Potential of Different Genotypes of *Ziziphus nummularia*, Their HPLC-UV, and Molecular Docking Analysis

**DOI:** 10.3390/molecules25215011

**Published:** 2020-10-29

**Authors:** Nisar Uddin, Niaz Ali, Zia Uddin, Nausheen Nazir, Muhammad Zahoor, Umer Rashid, Riaz Ullah, Ali S. Alqahtani, Abdulaziz M. Alqahtani, Fahd A. Nasr, Mengjun Liu, Mohammad Nisar

**Affiliations:** 1 Department of Botany, University of Malakand, Chakdara 18000, Dir (L), KPK, Pakistan; shallnr@yahoo.com (N.U.); nausheen.nazir@uom.edu.pk (N.N.); 2 Department of Botany, Hazara University Mansehra, Mansehra 21130, KPK, Pakistan; niazalitk25@gmail.com; 3 Research Center of Chinese Jujube, Hebei Agricultural University, Baoding 071001, China; lmj1234567@aliyun.com; 4 Department of Pharmacy, COMSATS University Islamabad, Abbottabad Campus, Abbottabad 22060, KPK, Pakistan; 5 Department of Biochemistry, University of Malakand, Chakdara 18000, Dir (L), KPK, Pakistan; mohammadzahoorus@yahoo.com; 6 Department of Chemistry, COMSATS University Islamabad, Abbottabad Campus, Abbottabad 22060, KPK, Pakistan; umerrashid@cuiatd.edu.pk; 7 Department of Pharmacognosy, (MAPPRC), College of Pharmacy, King Saud University, Riyadh 11451, Saudi Arabia; rullah@ksu.edu.sa (R.U.); alalqahtani@ksu.edu.sa (A.S.A.); 439106051@ksu.edu.sa (A.M.A.); fnasr@ksu.edu.sa (F.A.N.)

**Keywords:** cholinesterase inhibition, genotypes, medicinal uses, *Ziziphus nummularia* (Burm. f.), characterization, molecular docking, HPLC

## Abstract

*Ziziphus nummularia* is an important source of valuable phytoconstituents, which are widely used in traditional medicine system of Indo-Pak sub-continent. In this study we investigated the distribution of phenolic compounds in the fruit pericarps of six different genotypes (ZNP01-06) of *Z. nummularia* growing in the unexplored hilly areas of Pakistan. The methanolic extracts of these genotypes were screened for total phenolic content (TPC), total flavonoid content (TFC), antioxidant, and cholinesterase inhibitory potentials. The observed biological potentials were explained in terms of the outcome of molecular docking and HPLC analyses. Among them, genotype ZNP02 displayed high TPC (88.50 ± 1.23 μg/mL) and showed potent scavenging activity against DPPH (67.03 ± 1.04 μg/mL) and ABTS (65.3 ± 1.74 μg/mL) in comparison to ascorbic acid (68.7 ± 0.47 μg/mL). Moreover, genotypes ZNP01, ZNP02, and ZNP04 displayed potent inhibition against acetyl and butyryl cholinesterases (AChE and BChE) with IC_50_ values of 21.2, 20.5, and 23.7 μg/mL (AChE) and 22.7, 24.4, and 33.1 μg/mL (BChE), respectively. Furthermore, the individual compounds in the most potent species ZNP01 responsible for potent enzyme inhibition (identified through HPLC-UV analysis), were computed via docking simulation software to the enzyme structures. Among these compounds rutin exhibited significant binding affinity with value of −9.20 kcal/mol. The differences amongst the phytochemical compositions of the selected genotypes highlighted the genotypic variations in them. Based on our results it was concluded that the selected plant can be used as remedy of oxidative stress and neurodegenerative diseases. However, further studies are needed to isolate responsible compounds and test the observed potential in vivo, along with toxicological evaluations in animal models.

## 1. Introduction

Phenolic metabolites are biologically active compounds having great therapeutic potential. Many of these phytochemicals act as antioxidants by scavenging free radicals and, hence, exhibit therapeutic effects against several disorders caused by oxidative stress [1,2], for they are known as radical scavengers, metal chelators, reducing agents, hydrogen donors, and singlet oxygen quenchers [3]. Phenolic constituents present in plants have strong antioxidant potential and could protect living cells against oxidation-related damage [4,5], especially in the suppression of oxidative damage, which leads to several complications, such as cancer, chronic inflammation, dementia, and Alzheimer’s disease.

Alzheimer’s disease (AD) is a well-known neurodegenerative ailment that is characterized by gradual memory deficits which further impair cognitive functions [6,7]. More recently, AD has become a major public health concern in developed countries, and it is estimated to account for 50–60% of dementia cases in humans over 65 years of age [7,8]. In AD patients, deficiency has been observed in the concentrations of the neurotransmitter acetylcholine (ACh) in the brain. Inhibition of the acetyl cholinesterase (AChE) enzyme, which causes hydrolysis of ACh, is seen as a major treatment option for AD [9]. Traditionally used medicinal plants offer valuable alternative resources as enzyme inhibitors. Several natural-origin anticholinesterase antagonists have been reported, such as galantamine, being purified from the plants of Amaryllidaceae family, which is used to treat AD-related complications [10]. The AChE inhibitory effects of the given drug provide symptomatic relief by increasing the presence of ACh in the synapse by inhibiting AChE [11]. Similarly, butyrylcholinesterase (BChE) belonging to the cholinesterase family that hydrolyses different choline-based esters. Herbal products and natural phytochemicals like flavonoids, flavonols, and other bio-active compounds, exhibit health-promoting effects. Evidence suggests that these metabolites have the potential to protect different cells from oxidative damage [12,13].

*Ziziphus nummularia* (Burm. f.) Wight & Arn., is an edible small shrub of 6–8 feet high, belonging to family Rhamnaceae and found throughout Worthwestern India, Pakistan, and China [14]. *Ziziphus nummularia* is known by different local names such as Jharberi, Jadiaber, and Bhor, etc., with widespread uses as an edible stuff, particularly its fresh and dried fruits which are eaten and its juices consumed as a refreshing drink, as well as used in folk medicine [15,16]. *Z. nummularia* is a very important medicinal plant in the traditional system of the Indo-Pak subcontinent. The dried fruits of this plant contain alkaloids, saponins, and triterpenoids. Ethno-medicinally, *Z. nummularia* fruits are used for tis cooling, astringent, and appetizer effects, and in stomach-related problems [17]. Further, these fruits are used to treat various diseases, like chronic fatigue, diarrhea, bronchitis, burns, anemia, irritability, and pharyngitis, aid digestion, and in some areas it is used as a sedative, refrigerant, and tonic [18]. Similarly, this plant is employed in treating ulcers, cuts, and pulmonary diseases [19]. It has also been used in the treatment of fever and wound healing [20]. *Z. nummularia* is a rich source of phenolic compounds, has key roles in protecting cells against stress damage, and many of their biological activities are attributed to their cytotoxic and antioxidant potential [3,4,5]. To the best of our knowledge no study has been conducted to assess the cholinesterase inhibitory effects and antioxidant capacities of the different genotypes of *Z. nummularia* fruits found in different geographical locations in Pakistan, which could possibly have variations in the content of their phytochemical compositions.

The current work was aimed to evaluate the antioxidant and cholinesterase inhibitory potential of different genotypes of *Z. nummularia.* Different genotypes were collected from diverse agro-ecological zones of Pakistan, extracted with methanol and their total phenolic and flavonoid contents. Furthermore, the responsible compounds were identified through HPLC analysis and to support our observed results the compounds were docked with protein structures of AChE and BChE to determine the binding affinities in the binding pockets of enzymes.

## 2. Results and Discussion

### 2.1. Total Phenolic Content (TPC) and Total Flavonoid Content (TFC)

The fruits of *Z. nummularia* (locally known as wild Bera or Mall) are medicinally-important and nutritionally-rich sources of different metabolites having diverse beneficial effects and, hence, used for several purposes, such as cooling, appetizer, and stomachic effects [18]. Phenolic compounds are present in almost all plants and are the major bio-active constituents, having the ability of free radical scavenging and exhibit anti-oxidant activities. Thus, polyphenol rich food and edible materials are considered as beneficial to health and general well-being [21,22,23,24]. In the current study, methanol extracts of six different genotypes (ZNP01, ZNP02, ZNP03, ZNP04, ZNP05, and ZNP06) of the *Z. nummularia* fruit were collected from different geographical locations and were subjected to quantification of their total phenolic and total flavonoid contents. To estimate total flavonoids, gallic acid calibration curve was drawn using its different dilutions such as 20, 40, 60, 80, and 100 mg/mL. Results showed that the total phenolic contents of methanolic extract of ZNP01, ZNP02, ZNP03, ZNP04, ZNP05, and ZNP06 were 88.893 ± 1.353, 88.503 ± 1.231, 82.063 ± 2.069, 88.810 ± 1.336, 85.487 ± 0.386, and 85.777 ± 0.90 mg of gallic acid equivalent per 100 g (mg GAE/100 g) of dry sample. Among the different genotypes, we observed the highest TPC in genotype ZNP01, 88.893 ± 1.353 and ZNP02, 88.503 ± 1.231 mg GAE/100 g, rest of genotypes also exhibited significant TPC content which are given in Table 1. Moreover, a clear variation in the TPC and TFC content of the same specie but different genotypes highlighted the impact of different geographical locations very clearly (Table 1). Plants contains different phenolic compounds displaying various pharmacological effects, most of the therapeutic properties of the natural stuffs come from these phenolic constituents which play their role as anti-oxidants and quench the free radicals in the cellular environment [25,26].

Similarly, total flavonoid content in different genotypes were quantified using quercetin as a standard for the determination of total flavonoid content. A curve was established using different dilutions of quercetin such as 20, 40, 60, 80, and 100 µg/mL. Our results showed that the total flavonoid content of methanol extracts of ZNP01, ZNP02, ZNP03, ZNP04, ZNP05, and ZNP06 were 74.083 ± 0.601, 76.023 ± 0.974, 68.260 ± 1.584, 74.350 ± 0.743, 58.130 ± 1.041, and 63.350 ± 0.344 mg of quercetin equivalent per 100 g (mg QE/100 g) of dry sample, respectively (Table 1), and the highest TFC was shown by genotype ZNP01 (74.083 mg QE/100 g) ZNP02 (76.023 mg QE/100 g), and ZNP04 (74.350 mg QE/100 g). TPC represents the total phenolic content of an extract which also includes TFC and other phenolics, which has direct correlation with antioxidant potential of any extract. These results pointed out that significant difference exists in total phenolic contents of different genotypes, whereas total flavonoid content also displayed a matching trend as observed in the case of TPC. The variations in phytochemical compositions of these different genotypes clearly indicates the environmental effect on the production and concentration of secondary metabolites.

### 2.2. DPPH Radical Scavenging Capacity

**Figure 1:**
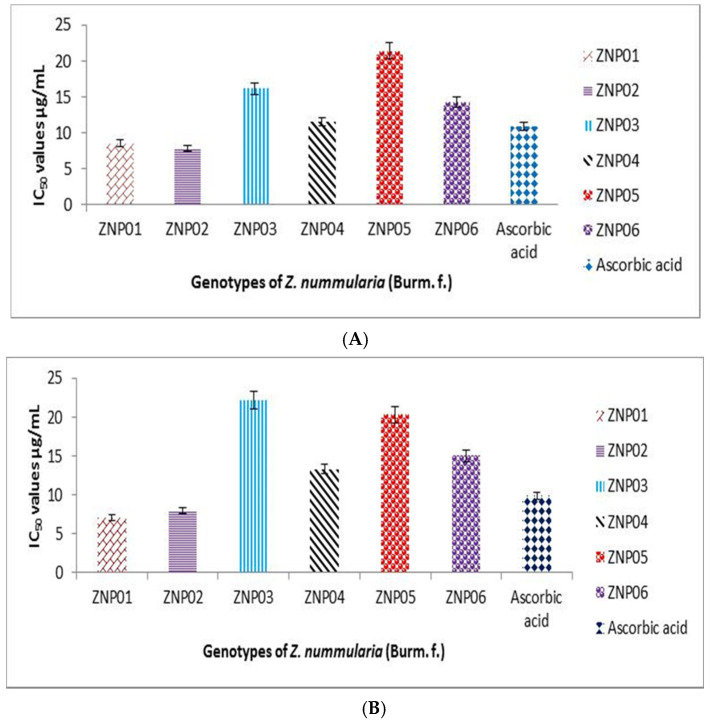
IC_50_ values of six different genotypes of *Z. nummularia* against DPPH (**A**) and ABTS (**B**).

Antioxidants are the substances which inhibits the oxidation caused by reactive oxygen species as they have the capability of scavenging these free radicals which are usually produced during metabolism. Several strategies are adopted for the measurement of antioxidant potential of any test sample, among them DPPH assay is frequently used to determine the free radical scavenging capacity of target compounds or extracts. DPPH radical scavenging assay was used to determine antioxidant potential the selected six genotypes of *Z. nummularia* (ZNP01, ZNP02, ZNP03, ZNP04, ZNP05, and ZNP06) [27,28]. The genotypes ZNP01, ZNP02, ZNP03, ZNP04, ZNP05, and ZNP06 exhibited 65.56 ± 1.56, 67.03 ± 1.04, 59.06± 0.58, 62.66 ± 0.28, 56.03 ± 0.53, and 62.77 ± 0.28 percent DPPH scavenging potential at lowest concentration (31.25 µg/mL), respectively. The IC_50_ values of all genotype are shown in Figure 1A. Ascorbic acid was used as a positive control which displayed inhibition of 68.70 ± 0.47% at lowest tested concentration (31.25 µg/mL) with 10.86 µg/mL of IC_50_ value (Figure 1 and Table S1). Previously a study has reported the anti-oxidant activity of *Z. nummularia,* but they have assessed the activity using a single genotype of this species which was grown and collected in India, that is totally different geographical location having different environmental conditions (hot and humid climate) [29]. However, in our study we have evaluated the activities of six different genotypes of *Z. nummularia* which were collected in northern areas of Pakistan having different climatic conditions. It is evident from our results that these environmental factors which ultimately affect the quality and quantity of secondary metabolites greatly contributes to the variance in content of bioactive compounds.

The scavenging capacities of these different genotypes were mainly due to the phenolic compounds present in respective genotypes. The observed trend in free radical scavenging capacities were related with concentrations of phenolic and flavonoid contents. A genotype with higher TPC and TFC exhibited higher anti-oxidation activities (Table 1 and Figure 1).

### 2.3. ABTS Radical Scavenging Capabilities

To further confirm the anti-oxidant potential of the selected genotypes were determined through another free radical assay; the ABTS assay [30]. Different dilutions such as 1000, 500, 250, 125, 62.5, and 31.25 µg/mL were mixed with methanol extracts of these genotypes. Genotypes ZNP01, ZNP02, ZNP03, ZNP04, ZNP05, and ZNP06 exhibited percent inhibition of 64.74 ± 1.15, 65.31 ± 1.74, 55.48 ± 0.76, 61.96 ± 0.29, 54.55 ± 0.32, and 61.88 ± 0.44% (Table S1). Among them, genotype ZNP02 showed highest % ABTS inhibition value of 65.31 ± 1.74 mg/mL. The IC_50_ values exhibited by these genotypes are shown in Figure 1. Ascorbic acid was used as a positive control that exhibited concentration dependent response of 66.43 ± 0.73 mg/mL at lowest concentration of 31.25 µg/mL with IC_50_ value of 9.88µg/mL (Figure 1). The observed antioxidant activities of *Z. nummularia* genotypes were due to variations in the content of phenolic compounds present in each genotype. It is evident from our results that ZNP01 and ZNP02 having higher TPC (ZNP01 = 74.083, ZNP02 = 76.023) and TFC (ZNP01 = 88.893, ZNP02 = 88.503) and, thus, have displayed potent anti-oxidant activities as compared to rest of genotypes (having low TPC and TFC content as well as lower anti-oxidant potential).

### 2.4. Acetyl Cholinesterase Inhibition Potential

To explore the AChE and BChE enzymes inhibitory potential of different genotypes of *Z. nummularia* fruits against AChE, a reported method was used with moderate modifications [31]. The extracts showed substantial activities against AChE and BChE which were confirmed by docking studies. The inhibitory potential was evaluated at various concentrations (1000, 500, 250, 125, 62.5, and 31.25 μg/mL). The genotypes ZNP01, ZNP02, ZNP03, ZNP04, ZNP05, and ZNP06 exhibited 55.54 ± 1.23, 52.78 ± 1.47, 49.12 ± 0.40, 51.04 ± 1.48, 55.42 ± 0.44, and 50.22 ± 2.76% AChE inhibition at the lowest tested concentration of 31.25 μg/mL, respectively.

**Figure 2:**
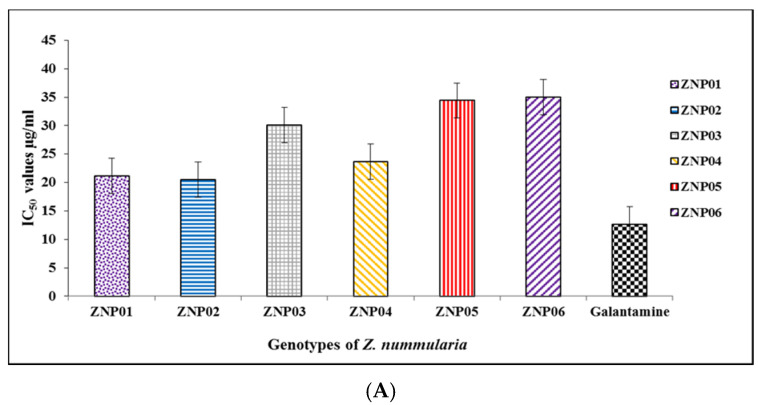

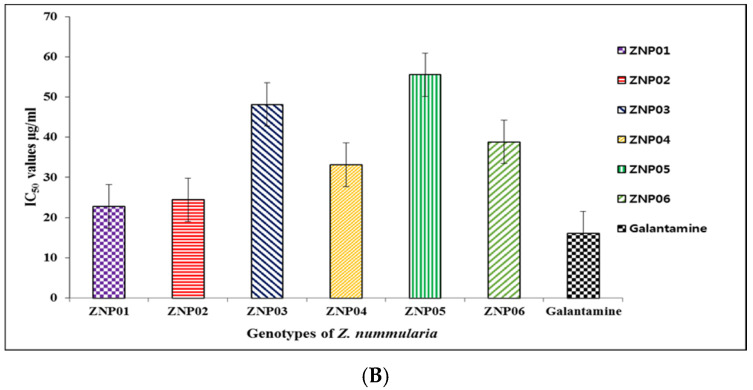
IC_50_ values of different genotypes of Z. *nummularia* of AChE (**A**) and BChE (**B**) inhibitory activities.

All genotypes displayed significant inhibitory effects, especially genotype ZNP01 having (IC_50_ = 21.19 μg/mL), ZNP02 (IC_50_ = 20.52 μg/mL) and ZNP04 (IC_50_ = 23.68 μg/mL) were the most potent genotypes among the tested samples. AChE inhibitory potential and IC_50_ values of all six genotypes of *Z. nummularia* are shown (Figure 2A and Table S2). Galantamine, used as a positive control displayed 61.42 ± 0.79% inhibition at 31.25 µg/mL with IC_50_ value 12.69 µg/mL (Figure 2A and Table S2). The potency of these genotypes indicated that they could be used as therapeutic agents against dementia and related diseases. Furthermore, the variation in the potencies of these genotypes is based on the quality and quantity of bioactive compounds present in them. The variation can be attributed to different needs of plants in different geographical locations.

### 2.5. Butyryl Cholinesterase Inhibition Potential

The butyryl cholinesterase inhibitory potential was tested for concentrations; 1000, 500, 250, 125, 62.5, and 32.25 μg/mL of each genotype. Genotype ZNP01, ZNP02, ZNP03, ZNP04, ZNP05, and ZNP06 exhibited 52.15 ± 1.36, 51.16 ± 1.16, 50.88 ± 0.28, 48.24 ± 0.43, 47.69 ± 1.16, and 45.74 ± 0.27 percent inhibitions at lowest concentration of 31.25 μg/mL (Table S2). Among them, genotype ZNP01 showed the highest BChE inhibition potential with IC_50_ = 22.76 μg/mL while ZNP02 exhibited inhibition with IC_50_ value of 24.44 μg/mL. Similarly, galantamine was used as a positive control that exhibited concentration dependent response with IC_50_ value 20.76 µg/mL (Figure 2B). As mentioned above, the beneficial effects of *Z. nummularia* fruit extracts are due to the variation in their bioactive constituents. Consistent with AChE inhibitory activities, these genotypes also exhibited the same pattern of variation in their potential against BChE that is linked with the differences in concentration of secondary metabolites in different genotypes (ZNP01-ZNP06) originated in them due to different needs in different geographical locations.

### 2.6. HPLC Characterization of Phenolic Compounds

**Figure 3:**
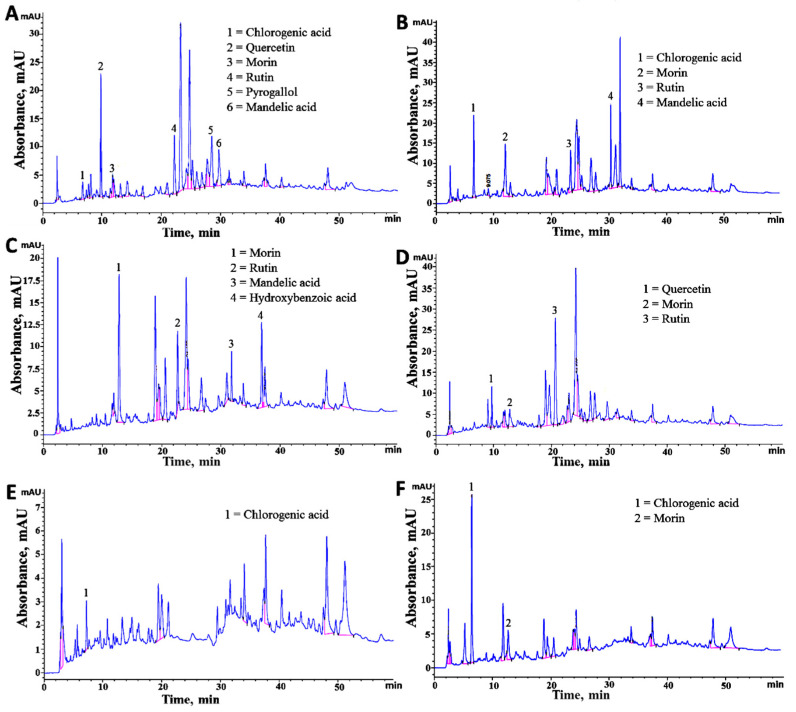
HPLC-UV chromatograms of different genotype of *Z. nummularia*: (**A**) ZNP01, (**B**) ZNP02, (**C**) ZNP03, (**D**) ZNP04, (**E**) ZNP05, and (**F**) ZNP06.

**Figure 4:**
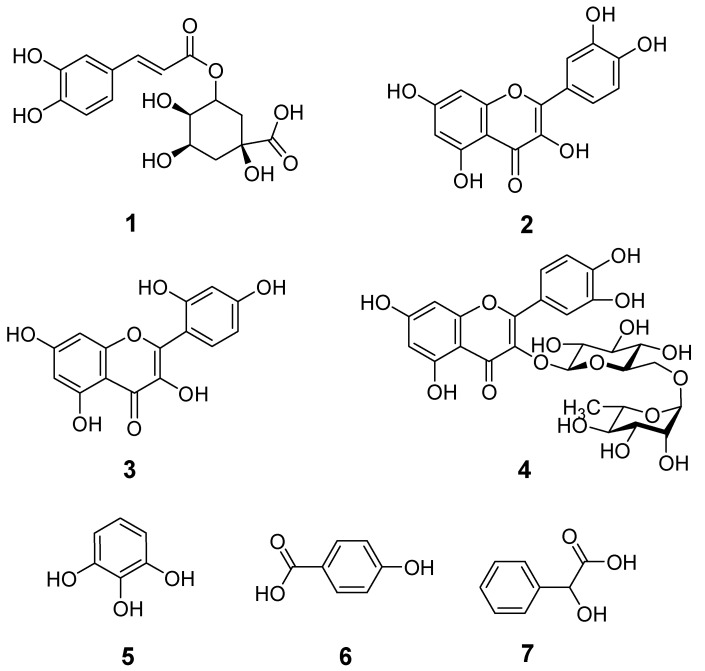
Chemical structures of the detected compounds.

To identify and quantify the bioactive compounds present in the different extracts of fruits, HPLC analysis of the targeted genotypes were performed. The bioactive compounds were identified by HPLC analysis through comparison with standard compounds (commercial compounds) and those reported in literature based on coincidences in retention time (Table 2 and Figure 3). Figure 3 presented the typical chromatograms of all the genotypes. The identified possible phenolic compounds along with their respective chromatogram peaks and retention times (min) are given in Table 2. The genotype ZNP01 contained chlorogenic acid, quercetin, morin, rutin, pyrogallol, and mandellic acid (Figure 4). In genotype ZNP02, four phenolic compounds chlorogenic acid, morin, rutin, and mandellic acid were identified. Phenolic compounds identified in genotype ZNP03 were morin, rutin, mandellic acid, and hydroxy benzoic acid. Quercetin, morin, and rutin were identified in genotype ZNP04 while chlorogenic acid was identified in genotype ZNP05. Similarly, in genotype ZNP06 morin and rutin were observed (Table 2). During the analysis qualitative variation was observed in different genotypes, in which ZNP01 consisted of maximum number of metabolites as compared to rest of genotypes as given in Table 2. Similarly, the least number of secondary metabolites were detected in genotypes ZNP04, ZNP05, and ZNP06. These results are in agreement with the observed TPC, TFC, anti-oxidant, and anti-cholinesterase activities.

Furthermore, we also calculated the content of individual metabolite present in different genotypes (ZNP01–ZNP06), our results indicated that appreciable amount of morin (ZNP01 − ZNP06 = 17.15 − 98.55 mg/100 g) and chlorogenic acid (1.36–13.12 mg/100 g) were present in the extracts of different genotypes, given in Table 2. Previous studies of *Z. nummularia* fruit extract has only revealed that different phenolic and other compounds are present in this plant, but they did not quantify the individual phenolic compounds [14]. Hence, several metabolites were identified in the fruit extracts such as morin, rutin, chlorogenic acid, mandellic acid, quercetin, pyrogallol, and hydroxy benzoic acid. Among them, morin was detected in all genotypes whose concentration was the highest (ZNP01 − ZNP06 = 17.15 − 98.55 mg/100 g) followed by chlorogenic acid, whereas low concentrations of rutin and quercetin were quantified from HPLC chromatograms. Based on the results it was concluded that *Z. nummularia* is a rich source of several bioactive constituents having great variation in the concentrations and compositions of secondary metabolites, hence this variation is attributed to different geographical localities.

### 2.7. Docking Studies

**Figure 5:**
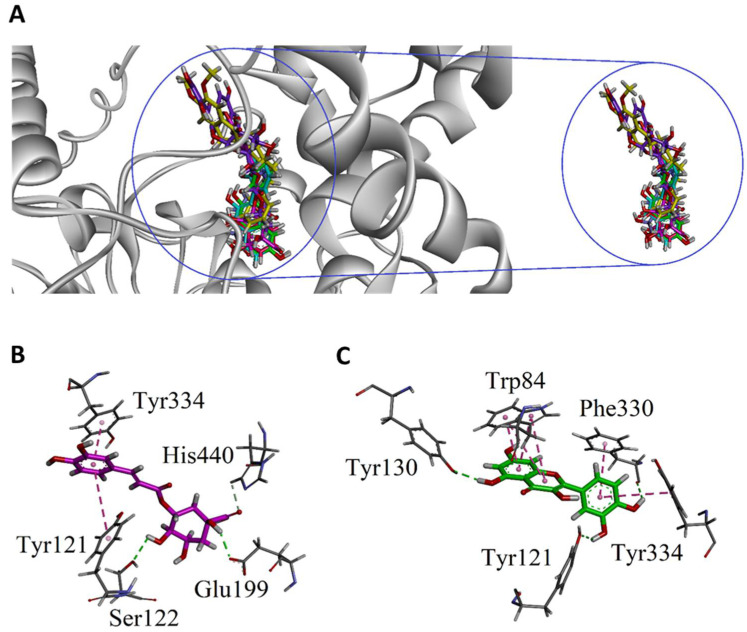
The overlaid ribbon diagram of all the constituents and native ligand donepezil (yellow) into the binding site of TcAChE (1EVE) (**A**). Close-up view interaction plots of the docked pose showing important residues with chlorogenic acid (**B**) and quercetin (**C**).

Docking studies of the reported bioactive constituents of the most potent genotype (ZNP01) of *Z. nummularia* was carried out using Molecular Operating Environment (MOE 2016.0802) software package. The purpose of the docking study was to investigate the role of each constituent in the ZNP01 species in inhibiting the targeted enzymes. For that purpose, receptor binding affinity values were considered as the criteria for contribution of each ligand in bioactivity. The crystal structure of Torpedo californica AChE (TcAChE, PDB code 1EVE) with native ligand donepezil was obtained from the Protein Data Bank. Docking algorithm was confirmed as reliable one by re-docking of the native ligand. The root mean square deviation (RMSD) computed was found 0.91 Å which is within the threshold limit (<2.0 Å). As shown in Figure 5A, all the bioactive compounds bind in the active site in a similar pattern like that of the native ligand donepezil.

Among the active constituents, the three-dimensional (3-D) binding interactions of the phenolic compounds such as chlorogenic acid and quercetin are shown in Figure 5B,C. Hydroxy groups of chlorogenic acid are engaged in hydrogen bond interactions with Glu199 and Ser122, while the phenyl group established π-π stacking interactions with two peripheral anionic site (PAS) residues Tyr121 and Tyr334 (Figure 5B). Quercetin formed three hydrogen bond interactions with one PAS residue (Tyr121) and two catalytic anionic site (CAS) residues Tyr130 and Phe330) via its hydroxyl groups (Figure 5C). Chromen-4-one forms a bifurcated π-π stacking interactions with important CAS residue Trp84. whereas dihydroxyphenyl group forms π-π stacking interactions with PAS residue Tyr334 (Figure 5C).

**Figure 6:**
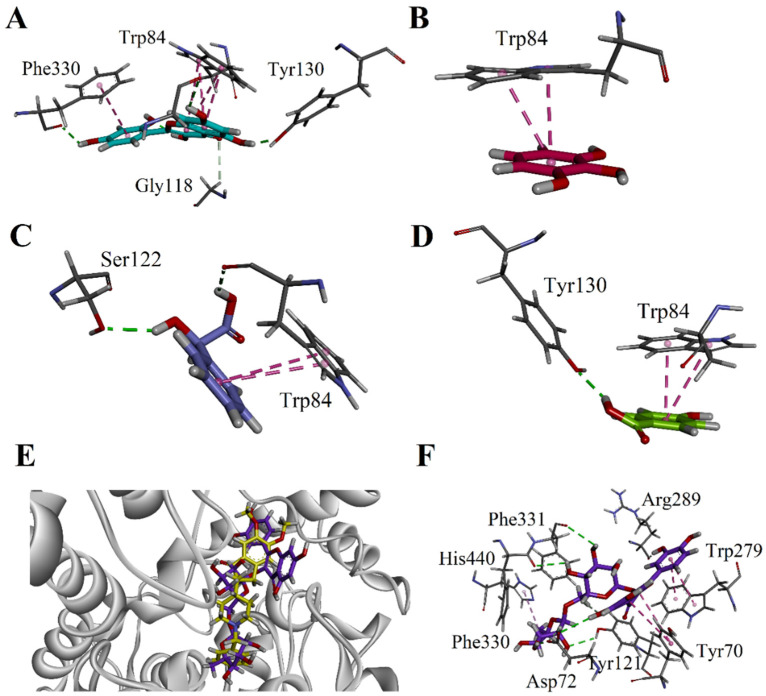
Close-up view interaction plots of the docked-pose showing important residues, morin (**A**), pyrogallol (**B**), mandelic acid (**C**), and hydroxybenzoic acid (**D**). The overlaid ribbon diagram of rutin and native ligand donepezil (yellow) into the binding site of TcAChE (1EVE) (**E**) and close-up depiction of the lowest-energy three-dimensional (3-D) docking poses of rutin (**F**).

Similarly, the 3-D binding interactions of other bioactive constituents present in the ZNP01 species such as morin, pyrogallol and mandelic acid are shown in Figure 6A–C. Morin forms three hydrogen bond interactions with Tyr130, Phe330 and Gly441. While chromen-4-one forms a bifurcated π-π stacking interactions with Trp84 (Figure 6A). Pyrogallol was able to show only π-π stacking interactions with Trp84 (Figure 6B), whereas mandelic acid established two hydrogen bonds and one π-π stacking interaction. Indole ring of Trp84 forms π-π stacking interaction with phenyl ring. While carbonyl oxygen of Trp84 forms hydrogen bond with -OH of carboxylic acid. The hydroxyl group of mandelic acid also forms hydrogen bond with Ser122 (Figure 6C). Hydroxy benzoic acid form π-π stacking interaction with Trp84. While hydroxy group ofTyr130 forms hydrogen bond interactions with the carboxylic group (Figure 6D).

Rutin is quercetin-3-*O*-rutinoside, the ribbon model of rutin superposed on native donepezil, and is shown in Figure 6D. The 3-D interaction plot showed that it forms a π-π stacking interaction with important PAS residue Tyr70 and Trp279. This suggests that it not only inhibits AChE hydrolysis but also Aβ-aggregation [25,26]. The rutin molecule also forms six hydrogen bond interactions with Asp72, Tyr121, Arg289, Phe330, and Phe331 (Figure 6E).

Furthermore, we were interested to analyze the synergistic effects of the identified bioactive compounds in terms of their binding affinities. Computed binding affinities are indicative of the ligand’s contribution and have great importance in identifying novel bioactive molecules. In the current study, rutin has the highest binding affinity value of −9.20 kcal/mol, followed by chlorogenic acid and quercetin with values of −7.06 and −6.46 kcal/mol, respectively. The computed binding affinity for pyrogallol and mandelic acid is −4.30 and −4.73 kcal/mol, respectively (Table 3). Based on the computed bioactivity data, we can conclude that rutin, chlorogenic acid, and quercetin may play important roles in the management of Alzheimer’s disease.

In natural products, hydroxyl groups are a common functionality. The presence of hydroxyl groups help the natural group to improve their water solubility and their derivatization. The derivatization may help in improving the AChE inhibition potential. A structure-activity relationship (SAR) study suggested that the maximum AChE inhibition depends upon the presence of hydroxyl groups. Phenyl rings are also determinants of the ligand-enzyme complex stability because of their aromatic-π stacking interactions with the key amino acid residues of PAS and CAS. Quercetin formed three hydrogen bond interactions via its hydroxyl groups, while it also forms two π-π stacking interactions with key residues of CAS and PAS. Rutin (quercetin-3-*O*-rutinoside) with more hydroxy groups forms five hydrogen bond interactions and two π-π stacking interactions. The stability of ligand-enzyme complexes is evident from computed binding energy values for both compounds (Table 3). Hence, we can conclude here that derivatization of identified compounds can be used to enhance the activity. Moreover, it has been reported that ligands interact with the peripheral anionic site (PAS) prevented AChE induced Aβ-aggregation, similarly it has been reported that AChE accelerates the nucleation of Aβ peptide in to Alzheimer fibrils by lowering peptide aggregation lag phase, therefore it has also been suggested that AChE play dual role by increasing the seeds required for nucleation and Alzheimer fibril elongation. All these consequences ultimately lead to Aβ-aggregation [32,33]. The 3-D interaction plot showed that rutin forms a π-π stacking interaction with important PAS residue Tyr70 and Trp279. Thus, it is assumed that apart from AChE inhibition, rutin may also prevent Aβ-aggregation. It is obvious from above binding energy data that these compounds (rutin, chlorogenic acid, quercetin, and morin) have the potential to inhibit acetylcholinesterase synergistically to prevent Alzheimer’s and other neurodegenerative diseases.

## 3. Material and Methods

### 3.1. Chemicals and Reagents

The 5,5-dithio-bis-nitrobenzoic acid (DTNB), electric eel acetylcholinesterase (type-VI-S), 2,2-diphenyl-1-picrylhydrazyl (DPPH), (Sigma-Aldrich, St. Louis, MO, USA), butyrylthiocholine iodide (Sigma-Aldrich, St. Louis, MO, USA), acetylthiocholine iodide (Sigma-Aldrich, London, UK), equine butyrylcholinesterase were obtained from Sigma-Aldrich (Waltham, MA, USA); ABTS or 2,2′-azinobis-3-ethylbenzothiazoline-6-sulfonic acid was obtained from Sigma-Aldrich (Darmstadt, Germany). Quercetin, morin, rutin, pyrogallol, phloroglucinol, hydroxy benzoic acid, chlorogenic acid, Folin–Ciocalteu, and HPLC-grade methanol were obtained from Sigma-Aldrich (London, UK) and used in the present study.

### 3.2. Plant Collection, Extraction and Fractionation

Fruits of *Z. nummularia* were gathered from diverse agro-ecological locations of Malakand, Khyber Pakhtunkhwa (KP), Pakistan. Mehboob ur Rahman, Govt. Post Graduate Jehanzeb College Swat, identified the fresh plants which were preserved in the Herbarium at University of Malakand, KP, Pakistan. Fruits were washed with distilled water; the pericarps were peeled with a sterilized blade and shade dried for enough time until complete removal of moisture. The dried pericarps were grinded into fine powder, and the weight was measured as 2 kg of powder materials which were macerated in methanol (80%) for 14 days. The filtrate obtained was subjected to a rotary evaporator that resulted in a solidified mass of 250 g, having an extraction yield of 12.5% [34].

### 3.3. Total Phenolic Content (TPC)

The total phenolic content of *Z. nummularia* was estimated following the protocol reported previously [35]. In a test tube, 100 µL of diluted extract, 500 µL of distilled water, and 100 µL of Folin–Ciocalteu reagent were added, mixed and left for 6 min, then 1000 µL of 7% sodium carbonate and again 500 µL of distilled water were added. Absorbance of mixture was measured at 760 nm using UV-Spectrophotometer after 90 min. The standard curve of gallic acid was obtained using dilutions (31.05, 62.5, 125, 250, 500, and 1000 µg/mL) for measuring TPC and was expressed as mg of gallic acid equivalent per gram of dry sample (mg GAE/g).

### 3.4. Total Flavonoid Content (TFC)

TFC of *Z. nummularia* fruits were assessed using the published procedure [36]. Quercetin was used as a standard and total flavonoid content was determined as milligram of quercetin equivalent per gram (mg QE/g) of dry sample. Calibration curve of quercetin was obtained using series of dilution (31.05, 62.5, 125, 250, 500, and 1000 µg/mL) prepared in methanol. About 500 µL of distilled water was mixed with 100 µL of each of dilutions and 100 µL of 5% sodium nitrate was mixed and left for 6 min then 150 µL of 10% solution of aluminum chloride was added and left for 5 min. To this mixture, 200 µL of 1 M sodium hydroxide was added and absorbance was recorded at 510 nm. A similar method in triplicate was repeated for all extracts.

### 3.5. DPPH Radical Scavenging Assay

DPPH assay was used to determine the antioxidant potential of extracts following previously reported method [37]. About 2 mg of DPPH was solubilized in 100 mL methanol to made DPPH stock solution. About 1 mg/mL solutions of the samples were prepared and diluted to concentrations of 1000, 500, 250, 125, and 62.5 μg/mL using methanol. A diluted solution of 0.1 mL was taken from every sample and mixed with 3 mL of DPPH solution. Incubation was done at 25 °C for 30 min and absorbance was recorded at 517 nm. The whole procedure was repeated in triplicate and the data acquired was presented as mean ± S.E.M. The % radical scavenging effects were calculated using the given Equation (1):

(1) $$\%\;\mathrm{Free}\;\mathrm{radical}\;\mathrm{scavenging}=\frac{\mathrm{Control}\;\mathrm{absorbance}-\mathrm{Sample}\;\mathrm{absorbance}}{\mathrm{Controlabsorbace}}\times 100$$

### 3.6. ABTS Free Radical Scavenging Assay

Antioxidant capacities of *Z. nummularia* fruit crude methanolic extract against the ABTS (2,2-azinobis [3-ethylbenzthiazoline]-6-sulfonic acid) radical were also assessed following standard protocol [38]. Solutions of potassium per-sulfate (2.45 mM) and ABTS (7 mM) were prepared. Absorbance of the ABTS solution was adjusted to 0.7 at 745 nm with the help of 50% methanol. Afterwards, 3 mL of ABTS solution and 300 μL of samples were mixed and their absorbance were noted at 745 nm for 6 min. The data was recorded in triplicate while the positive control used was ascorbic acid. The % ABTS free radicals scavenging was recorded using Equation (1).

### 3.7. Anticholinesterase Assay

*Z. nummularia* fruits extracts were assessed for their ability to inhibit BChE and AChE with the help of spectrophotometer employing the in-practice method [39]. Butyrylcholine iodide and acetylcholine iodide were used as substrate. About 5 μL BChE (0.01 U/mL) and AChE (0.03 U/mL) were taken in cuvette, DTNB (5 μL) and plant samples (205 μL of 125–1000 μg/mL dilutions) were mixed and transferred to water bath at 30 °C and incubated for 15 min. A spectrophotometer at wavelength 412 nm was used to check absorbance. Galantamine was used as positive control. Percent inhibition and percent enzyme activity was found by using following Equation (2):

(2) $$\%\;\mathrm{Inhibition}=\frac{\;\mathrm{Contr}\;\mathrm{Control}\;\mathrm{absorbance}-\mathrm{Sample}\;\mathrm{absorbance}}{\mathrm{Controlabsorbace}}\times 100$$

IC_50_ values of AChE and BChE were calculated with linear regression analysis using concentration versus their % inhibitory activities.

### 3.8. Samples Preparation and HPLC-UV Analysis

For HPLC analyses, 1 g of pericarp powder of six genotypes were added to 20 mL of water and methanol (1:1) in separate tubes, while gentle heat was provided to the samples in water bath at 50 °C for 1 h. Centrifugation of sample was done for 10 min at 4000 rpm and filtered through Whatman filter paper in labeled HPLC vials. Phytochemicals quantification were done using a high-performance liquid chromatography (HPLC) system (Agilent 1260 Infinity) having essential parts such as an auto-sampler, quaternary pump, degasser, and Agilent Zorbax Eclipse XDB-C18 column. The gradient system comprised of solvent B (acetic acid: methanol: deionized water, 20:100:180, *v*/*v*) and solvent C (acetic acid: deionized water: methanol, 20:80:900, *v*/*v*). Elution was performed at gradient flow, starting from 100% solvent B at 0 min, 85% solvent B at 5 min, 50% solvent B at 20 min, 30% solvent B at 25 min, and 100% solvent C from 30–40 min. The UV detector was set at 310 nm. The concentration of identified compounds were quantified using linear regression analysis.

### 3.9. Docking Studies

Docking studies were executed on Molecular Operating Environment (MOE 2016.0802) suite [40]. The crystal structure of Torpedo californica AChE (TcAChE, PDB code 1EVE) with native ligand donepezil, was obtained from the Protein Data Bank. Preparation of ligands and downloaded enzyme, active site identification, and the procedure of docking was executed by formerly reported methods [41,42]. The three-dimensional interaction plot of the drug-enzyme complex was analyzed using Discovery Studio Visualizer [43].

### 3.10. Statistical Analysis

The results were expressed as mean ± standard deviation. Statistical comparisons among all groups were performed by using ANOVA.

## 4. Conclusions

The current investigation reported the phenolic and flavonoid contents of the methanol extract of fruits of *Z. nummularia* genotypes, in which genotype ZNP01 showed highest level of total phenolic contents (88.89 mg GAE/100 g), whereas ZNP02 displayed highest level of total flavonoid content (76.023 mg QE/100 g). The genotype ZNP02 showed highest inhibition DPPH and ABTS radicals. The genotype ZNP03 and ZNP05 displayed strong inhibition of AChE and BChE with IC_50_ values 20.5 and 21.2 μg/mL, respectively. Furthermore, HPLC-UV analysis of different genotypes revealed the presence of phenolic compounds with variations from one another. In silico effects of all phyto-constituents were studied via molecular docking simulations. Types of interactions and binding affinities data suggested that rutin exhibited significant binding energy. Being an important medicinal plant, *Z. nummularia* fruit extract is a valuable source of potent bioactive compounds and could be used as effective therapeutic remedy against oxidative stress and neurodegenerative diseases. Further evaluation, including in vivo study, is required, particularly to isolate the pharmaceutically important metabolites responsible for the observed biological effects.

## Figures and Tables

**Table 1:** Total phenolic content (TPC) and total flavonoids content (TFC) in crude methanol extracts of different genotypes of *Z. nummularia.*

Samples	TPC (mg GAE/100 g)	TFC (mg QE/100 g)
ZNP01	88.893 ± 1.353	74.083 ± 0.601
ZNP02	88.503 ± 1.231	76.023 ± 0.974
ZNP03	82.063 ± 2.069	68.260 ± 1.584
ZNP04	88.810 ± 1.336	74.350 ± 0.743
ZNP05	85.487 ± 0.386	58.130 ± 1.041
ZNP06	85.777 ± 0.909	63.350 ± 0.344

**Table 2:** Characterization and quantification of phenolic compounds in different genotypes of *Z. nummularia* (Burm. f.) crude methanol extract by HPLC analysis.

Sample	No. of Peak	Retention Time (min)	Identified Phenolic Compounds	λmax (nm)	Peak Area of Sample	Peak Area of Standard	Con. (mg/100 g)
**ZNP01**	**1**	6.6	Chlorogenic acid	320	35.00	12.93	2.43
	**2**	9.8	Quercetin	320	208.70	7089.28	0.03
	**3**	12.5	Morin	320	38.31	2.01	17.15
	**4**	22.3	Rutin	320	68.22	2241.22	0.01
	**5**	28.5	Pyrogallol	320	182.25	1.02	161.76
	**6**	29.7	Mandelic acid	320	116.76	7195.52	0.02
**ZNP02**	**1**	6.6	Chlorogenic acid	320	188.48	12.93	13.12
	**2**	12.5	Morin	320	55.70	2.01	24.93
	**3**	22.3	Rutin	320	67.21	2241.22	0.01
	**4**	29.7	Mandelic acid	320	50.94	7195.52	0.01
**ZNP03**	**1**	12.5	Morin	320	220.18	2.01	98.55
	**2**	22.3	Rutin	320	28.30	2241.22	0.01
	**3**	29.7	Mandelic acid	320	54.69	7195.52	0.01
	**4**	36.9	Hydroxy benzoic acid	320	49.69	40.20	1.12
**ZNP04**	**1**	9.8	Quercetin	320	21.93	7089.29	0.002
	**2**	12.5	Morin	320	86.05	2.01	38.51
	**3**	22.3	Rutin	320	50.15	2241.22	0.01
**ZNP05**	**1**	6.6	Chlorogenic acid	320	19.51	12.93	1.36
**ZNP06**	**1**	12.5	Morin	320	35.81	2.01	16.03
	**2**	22.3	Rutin	320	39.08	2241.22	0.01

**Table 3:** Binding energy calculations of the identified phenolic compounds.

Identified Phenolic Compounds	Binding Energy (kcal/mol)
Chlorogenic acid	−7.06
Quercetin	−6.46
Morin	−6.26
Rutin	−9.20
Pyrogallol	−4.3
Mandelic acid	−4.73
Hydroxy benzoic acid	−4.59

## Supplementary Materials

The following are available online. Table S1: DPPH and ABTS free radical scavenging activities of different genotypes of Z. *nummularia*, Table S2: AChE and BChE inhibitory potentials of different genotypes of *Z. nummularia*.
