# Supplementary material for: Evaluation of Cholinesterase Inhibitory Potential of Different Genotypes of Ziziphus nummularia, Their HPLC-UV, and Molecular Docking Analysis
Source: Molecules. 2020 Oct 29;25(21):5011. doi: 10.3390/molecules25215011 (PMC7663671; doi:10.3390/molecules25215011)
Supplement: Supplementary file 1 [file molecules-25-05011-s001.pdf]

Table S1. Percent DPPH and ABTS free radical scavenging activity of genotypes of Z. *nummularia* at various concentrations

S.No	Genotypes	Concentration (µg/mL)	% DPPH Scavenging Mean ± SEM	IC_{50} µg/ml	% ABTS Scavenging Mean ± SEM	IC_{50} µg/ml
		250	84.62 ± 0.47		77.38 ± 1.41	
1		125	73.37 ± 0.48	8.57	73.07 ± 0.18	7.08
	**ZNP01**	62.5	68.68 ± 0.76		68.68 ± 0.76	
		31.25	64.74 ± 1.15		63.74 ± 1.15	
		15.625	57.31 ± 1.02		55.21 ± 0.76	
		7.812	49.05 ± 0.62		50.03 ± 1.10	
		250	83.00 ± 0.51		81.77 ± 0.48	
2	**ZNP02**	125	78.96 ± 1.22	7.79	76.59 ± 0.59	7.93
		62.5	73.70 ± 0.35		71.49 ± 0.42	
		31.25	67.03 ± 1.04		65.31 ± 1.74	
		15.625	56.21 ± 1.13		54.03 ± 0.84	
		7.812	49.56 ± 0.92		48.87 ± 1.89	
		250	78.37 ± 0.23		73.93 ± 0.96	
3	**ZNP03**	125	72.25 ± 0.86	16.22	65.09 ± 0.58	22.18
		62.5	65.29 ± 1.03		61.43 ± 1.39	
		31.05	59.06 ± 0.58		55.48 ± 0.76	
		15.625	48.40 ± 1.09		46.21 ± 0.52	
		250	80.99 ± 1.02		78.97 ± 0.48	
4	**ZNP04**	125	74.71 ± 0.14	11.52	72.10 ± 0.58	13.35
		62.5	69.26 ± 0.13		66.16 ± 1.43	
		31.05	62.66 ± 0.28		61.96 ± 0.29	
		15.625	52.78 ± 1.37		51.87 ± 0.18	
		7.812	42.07 ± 0.76		43.15 ± 0.21	

5	**ZNP05**	250	75.24 ± 1.28	21.48	71.28 ± 0.22	20.34
		125	68.36 ± 0.83		66.07 ± 1.07	
		62.5	62.22 ± 0.16		61.11 ± 1.98	
		31.05	56.03 ± 0.53		54.55 ± 0.32	
		15.625	45.09 ± 1.13		46.52 ± 0.64	
6	**ZNP06**	250	80.86 ± 0.57	7.79	79.70 ± 0.93	15.05
		125	75.87 ± 1.05		74.94 ± 0.28	
		62.5	68.23 ± 0.11		66.56 ± 0.38	
		31.25	62.77 ± 0.28		61.88 ± 0.44	
		15.625	55.68 ± 1.02		50.77 ± 0.57	
		7.812	50.17 ± 0.71		41.68 ± 0.28	
7	**Ascorbic acid**	250	85.96 ± 0.29	10.86	82.91 ± 0.46	9.88
		125	79.10 ± 0.57		76.74 ± 0.49	
		62.5	73.48 ± 0.76		71.61 ± 0.81	
		31.25	68.70 ± 0.47		66.43 ± 0.73	
		15.625	53.19 ± 1.09		55.50 ± 0.60	
		7.812	47.07 ± 0.98		46.07 ± 0.66	

Table S2. AChE and BChE inhibitory potentials of different genotypes of *Z. nummularia* at various concentrations

S.No	Genotypes	Concentration (μg/mL)	% AChE Scavenging Mean ± SEM	AChE IC_{50} (μg/mL)	% BChE Scavenging Mean ± SEM	BChE IC_{50} (μg/mL)
		500	82.68 ± 0.93		79.57 ± 0.60	
1		250	76.03 ± 0.31	21.19	73.54 ± 0.14	22.76
	**ZNP01**	125	69.83 ± 0.45		66.69 ± 0.07	
		62.5	62.77 ± 1.52		61.55 ± 0.53	
		31.25	55.42 ± 0.44		52.15 ± 1.36	
		15.625	45.57 ± 0.72		46.42 ± 1.08	
		500	74.78 ± 0.38		71.42 ± 0.43	
2	**ZNP02**	250	67.55 ± 0.34	20.52	64.77 ± 1.52	24.54
		125	62.83 ± 0.29		60.68 ± 0.12	
		62.5	57.57 ± 1.43		56.48 ± 0.11	
		31.25	53.12 ± 0.40		52.16 ± 1.16	
		15.625	46.12 ± 0.62		45.03 ± 1.31	
		500	75.12 ± 1.09		76.54 ± 0.18	
3	**ZNP03**	250	69.15 ± 0.31	30.07	71.59 ± 0.50	48.16
		125	63.95 ± 0.72		64.54 ± 1.35	
		62.5	57.36 ± 0.51		59.21 ± 0.34	
		31.25	51.04 ± 1.48		45.88 ± 0.28	
		1000	84.55 ± 1.55		78.79 ± 0.17	
4	**ZNP04**	500	76.52 ± 0.73	23.68	72.48 ± 0.78	33.13
		250	69.16 ± 0.55		67.86 ± 1.43	
		125	62.12 ± 0.34		61.62 ± 0.37	
		62.5	56.99 ± 0.31		55.10 ± 0.40	
		31.25	52.22 ± 2.76		48.24 ± 0.43	

5	**ZNP05**	1000	85.37 ± 0.08	34.42	81.62 ± 0.79	55.44
		500	79.65 ± 0.44		75.27 ± 2.88	
		250	72.35 ± 1.42		70.96 ± 0.09	
		125	65.21 ± 0.39		66.76 ± 0.80	
		62.5	59.66 ± 0.78		57.40 ± 0.04	
		31.25	48.54 ± 1.23		43.69 ± 1.16	
6	**ZNP06**	1000	81.24 ± 0.15	35.01	76.66 ± 1.50	38.82
		500	75.74 ± 1.18		69.98 ± 0.44	
		250	71.55 ± 0.68		63.54 ± 1.35	
		125	65.89 ± 0.51		56.36 ± 0.32	
		62.5	58.26 ± 0.34		51.01 ± 1.29	
		31.25	49.78 ± 1.47		46.74 ± 0.27	
7	**Galantamine**	500	85.74 ± 0.27	12.69	79.80 ± 0 36	20.76
		250	79.68 ± 0.18		73.65 ± 0 28	
		125	73.49 ± 0.20		65.42 ± 0.79	
		62.5	67.66 ± 0.35		61.68 ± 0.07	
		31.25	61.42 ± 0.79		55.69 ± 0.12	
		15.625	53.70 ± 0.25		48.42 ± 0.31	
